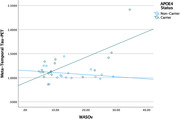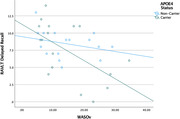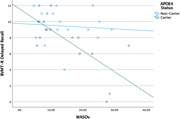# Variable Sleep Quality Is Related to Tau Burden and Worse Memory in Older Women at Risk for Alzheimer’s Disease

**DOI:** 10.1002/alz.090079

**Published:** 2025-01-03

**Authors:** Jordan Stiver, Xin Wang, Kitty K. Lui, Melanie A Dratva, Nadine Heyworth, Judy Pa, Atul Malhotra, Erin E. Sundermann, Sarah Banks

**Affiliations:** ^1^ UC San Diego Health, La Jolla, CA USA; ^2^ University of California, San Diego, La Jolla, CA USA; ^3^ SDSU / UC San Diego Joint Doctoral Program in Clinical Psychology, San Diego, CA USA

## Abstract

**Background:**

Women are more likely to experience sleep problems than men, especially during and after menopausal transition. Sleep disturbances are related to memory decline and the development of Alzheimer’s disease (AD), which is also more common among women. While research on habitual sleep patterns in aging has largely focused on mean sleep outcomes across nights, few studies have examined the potentially harmful effects of night‐to‐night variability in sleep quality on AD biomarkers and memory function. The purpose of this study was to determine how intraindividual variability in nocturnal sleep quality relates to tau burden and memory function in older women at elevated genetic risk for AD.

**Methods:**

Older women (*N* = 33; *M*_age_ = 73.1, *SD* = 4.0; APOE‐ε4 carriers, *n* = 17) with mild cognitive impairment and an AD polygenic hazard score ≥50%ile wore an actigraphic device on the wrist for an average of 7.6 nights (range = 5‐13) to objectively measure sleep behavior. Participants also completed neuropsychological tests and ^18^F‐MK6240 tau‐PET to quantify tau burden. Variability in wake time after sleep onset (WASOv) was calculated as the intraindividual standard deviation of WASO across sleep intervals. Memory was assessed using delayed recall scores from the Logical Memory (LM) subtest of the Wechsler Memory Scale‐Revised, the Rey Auditory Verbal Learning Test (RAVLT), and the Brief Visuospatial Memory Test‐Revised (BVMT‐R). A composite tau‐PET temporal meta‐region of interest was calculated based on standardized uptake value ratios using inferior cerebellar gray matter as the reference region.

**Results:**

After controlling for age, APOE‐ε4 carrier status, and mean WASO, regression models showed that WASOv predicted greater meta‐temporal tau‐PET signal (β = .52, *p*<.05) and worse delayed recall on LM and BVMT‐R (β = ‐.58 and β = ‐.63, respectively; *p*s<.01). Results further showed interaction effects, such that WASOv was associated with greater tau as well as worse RAVLT and BVMT‐R performances, especially among APOE‐ε4 carriers (|β| range = .67–.89, all *p*s<.05).

**Conclusions:**

Variability in sleep quality is associated with greater tau burden and worse memory function in older women at risk for AD, particularly among APOE‐ε4 carriers. Results highlight the possibility that variable sleep quality is a modifiable risk factor in the early pathogenesis of AD and its cognitive sequelae.